# A literature review of remote mental health screening: barriers, potential solutions, and tools

**DOI:** 10.3389/fdgth.2025.1670691

**Published:** 2025-10-13

**Authors:** Nicole Quiram, Tamjid Salam, Fatima Sadjadpour, Niyousha Hosseinichimeh, Lenore Jarvis, Lamia Soghier

**Affiliations:** ^1^ Grado Department of Industrial and Systems Engineering, Virginia Polytechnic Institute and State University, Blacksburg, VA, United States; ^2^ Department of Mechanical & Industrial Engineering, The University of Illinois Chicago, Chicago, IL, United States; ^3^ Division of Emergency Medicine, Children’s National Hospital, Washington, DC, United States; ^4^ School of Medicine and Health Sciences, Geroge Washington University, Washington, DC, United States; ^5^ Department of Neonatology, Children’s National Hospital, Washington, DC, United States

**Keywords:** remote measurement technologies (RMTs), barriers of implementing RMTs, solutions to enhance use of RMTs, assessment surveys, mental health

## Abstract

Remote Measurement Technologies (RMTs) have the potential to become widely used tools for monitoring and treating mental health. However, their adoption faces multiple barriers. We conducted a focused literature review to identify commonly used devices and assessment surveys, synthesize barriers to their use, and explore proposed solutions. Our review highlighted several challenges in implementing RMTs, including technological limitations, user-related factors, legal and ethical concerns, research constraints, and difficulties integrating these technologies into clinical practice. While studies have examined barriers through user interviews, empirical analyses of success factors remain limited, highlighting a need for further research in this area.

## 1 Background

Remote Measurement Technologies (RMTs) are increasingly utilized for screening, monitoring, and treatment of mental health conditions. Remote Measurement Technologies (RMTs) are digital tools—such as smartphones, wearables, and associated apps—that collect data in real time either passively or actively (1). Passive data collection involves gathering information through embedded sensors or user interactions with the device, such as tracking steps via an accelerometer or measuring heart rate using photoplethysmography—a technique that uses light, typically green LEDs, to detect changes in blood volume beneath the skin. Active data collection, on the other hand, requires direct input from individuals, such as completing mood surveys or logging medication use through a smartphone app or web link.

Clinicians are increasingly seeking ways to access hard to reach populations such as rural areas, low-income communities, and postpartum caregivers. RMT provides clinicians with further reach than traditional means. For example, at Children's National in Washington DC, a Level IV Neonatal Intensive Care Unit (NICU), caregivers of children are often not present at the bedside. The infants are transported to the hospital from over 40 area NICUs and parents often return to work during their infant's long length of stay. Screening and treatment of these postpartum caregivers is hampered by absence from the bedside. Therefore, we are frequently looking for RMT solutions to overcome this problem.

Although the clinical utility of RMTs in improving depression symptoms and outcomes is yet to be determined (1–3), these technologies provide multiple other benefits. First, RMT can increase access to healthcare (4) and overall capacity of mental healthcare. Many individuals experience barriers to traditional in-person care, due to geographic distance, limited mobility, or the stigma associated with seeking help for mental health (5). By using RMTs, healthcare providers can reach patients who might otherwise be excluded from timely, quality mental health support. However, health inequalities can persist if individuals with limited access, experience, or capacity are not equipped to benefit from these technologies (6). Second, continuous data collection from RMTs enables healthcare professionals to gather more accurate and detailed information about a patient's condition, rather than relying solely on episodic visits or self-reports (7). Thus, it helps to create a clearer picture of the patient's health at various points in time, enhancing the overall understanding of their condition without creating a large burden on the patient (7). Third, RMTs can assist in personalized detection of mental health symptoms. For example, data collected from mobile phones and sensors such as movement patterns and communication behaviors improve prediction of depressive symptoms (8). Finally, RMTs provide cost savings for both patients (i.e., transportation costs) and providers (i.e., facilities, and staffing) (5, 9, 10) and lead to reduced stress for patients (9).

Past systematic literature reviews of RMTs examined the impact of these technologies on depression outcomes. Goldberg and colleagues identified 13 randomized controlled trials, but only three specifically isolated the clinical impact of RMTs (2). One of these trials found that adding a monthly remote measurement-based care via secure messaging to treatment as usual (TAU) in primary care led to significantly greater symptom improvement compared to TAU alone. This systematic review concluded that while RMTs are feasible, further research is needed to assess their impact on depression outcomes (2). Another systematic review found that data derived from remote measurement technologies can be used to construct digital phenotypes that facilitate clinical assessment and may serve as predictors of relapse or symptom worsening (11). Walsh and colleagues conducted a realist review of RMTs for depression in individuals aged 14–24 years. They found that RMTs were useful for detecting changes in sleep, mobility, smartphone use, social communication, and mood, which supported screening, self-monitoring, and feedback to healthcare providers. However, RMTs were less effective for relapse prevention and delivering personalized interventions (1). Although past reviews investigated the context in which RMTs are useful, they have not synthesized the barriers of implementing RMTs for mental health problems and the proposed solutions. This study reviews the literature on RMTs to explore common devices and assessment surveys, identify barriers to their use, and examine solutions proposed to address these challenges.

## 2 Method

### 2.1 Search strategy

We conducted a literature review to identify journal publications about remote screening of mental health. The search was performed across three databases—PubMed, APA PsycNET, and Google Scholar—covering publications from 2004 to 2024. The table below outlines the keywords and search categories used for each database (Table 1).

**Table 1:** Database keywords and search categories.

Database	Keywords	Search category
PubMed	(remote screening) OR (remote measurement) OR	Title/Abstract
	(remote assessment) AND (mental health)	
	OR (depression)	
APA PsycNE	(remote screening) OR (remote measurement) OR	Title
	(remote assessment) AND (mental health) OR (depression)	
Google Scholar	(remote screening, mental health)	Separate search terms all in title
	(remote measurement, mental health)	
	(remote assessment, mental health)	
	(remote screening, depression)	
	(remote measurement, depression)	
	(remote assessment, depression)	

### 2.2 Inclusion criteria

**Figure 1:**
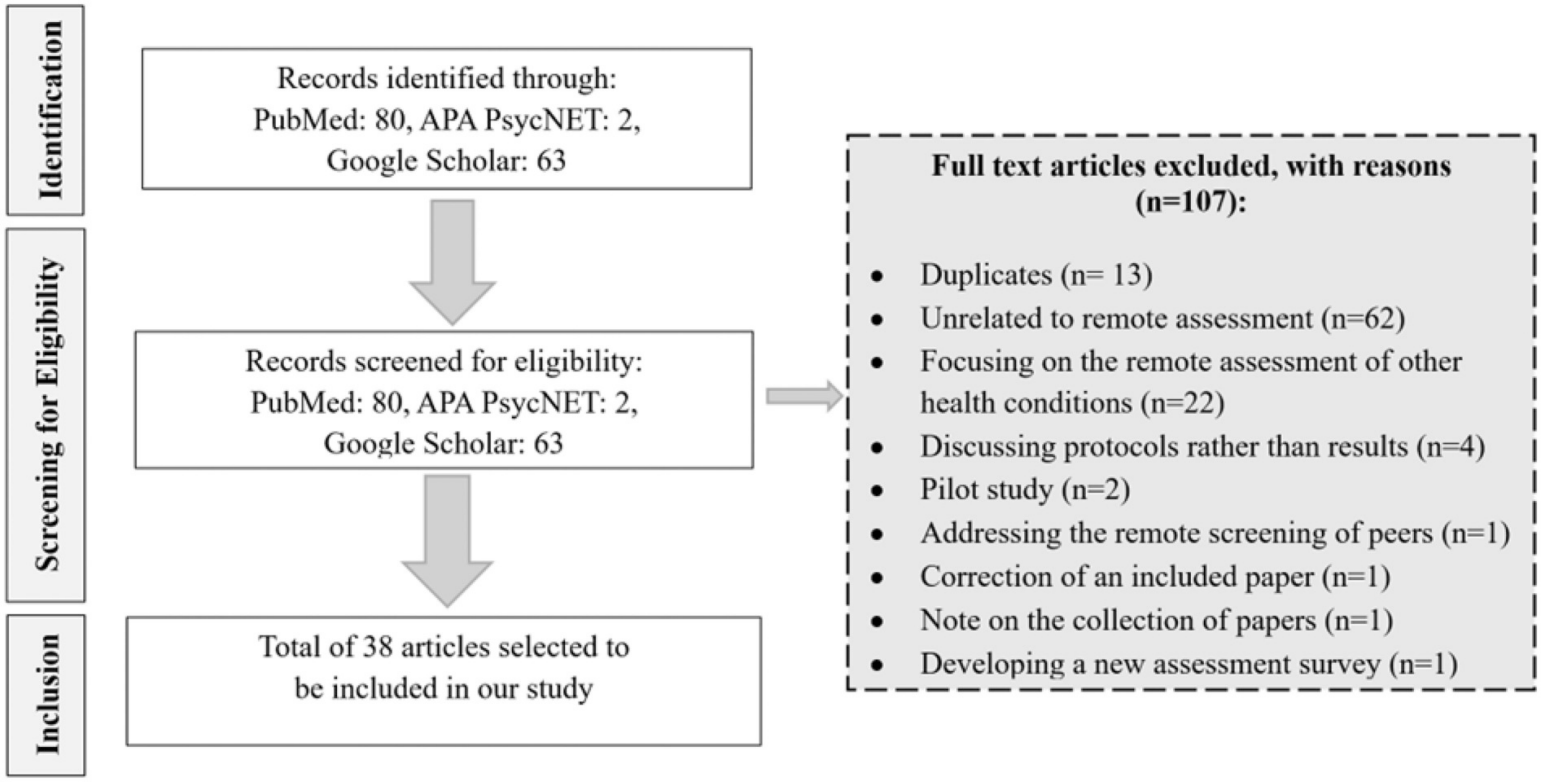
Flow diagram of the literature review process.

After conducting an initial search across three databases, we identified 80 papers in PubMed, 2 in APA PsycNET, and 63 in Google Scholar. The titles and abstracts of these 145 articles were screened, and duplicates and studies unrelated to remote screening for mental health and depression were excluded from further review. The exclusion criteria included the following: duplicates (*n* = 13), studies unrelated to remote assessment (*n* = 62), studies focusing on the remote assessment of other health conditions (*n* = 22), studies discussing protocols rather than results (*n* = 4), pilot studies (*n* = 2), studies addressing the remote screening of peers instead of individuals with mental health concerns (*n* = 1), a correction of an included paper (*n* = 1), a note on the collection of papers about mental health remote screening (*n* = 1), and developing a new assessment survey (*n* = 1) (Figure 1).

### 2.3 Study selection

After screening the abstracts retrieved from PubMed, APA PsycNET, and Google Scholar, we selected 38 studies for inclusion in our analysis. Through a detailed review of these papers, we identified the tools and assessment surveys used in remote screening, the barriers and success factors in implementing remote screening systems, and the pathways for connecting at-risk individuals to providers. A summary of the key steps and findings from this process is provided below.

## 3 Results

Results from the articles were grouped into 4 categories: (a) devices used, (b) assessment surveys utilized, (c) Strategies to connect patients to services, and (d) Barriers and Facilitators of RMT.

### 3.1 Devices used

**Figure 2:**
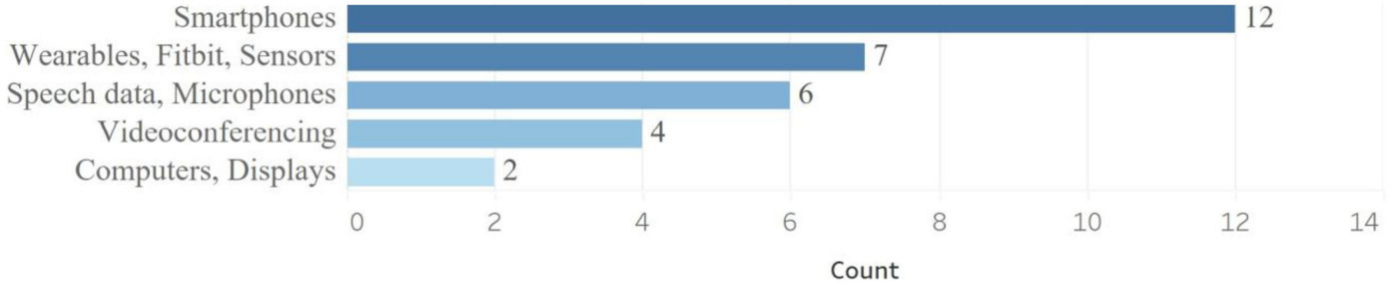
Devices used to collect data in studies of RMTs.

Multiple devices have been used to collect data, including smartphones (12–23), wearables (15, 17, 18, 21–24), computers/displays (10, 13, 19), videoconferencing (21, 25–27), and microphone of computers, phones, or tablet to record patients' speech (15, 18, 20, 24, 28, 29). As shown in Figure 2, the most common device used was smartphones.

Studies' objectives determine what device to use (1). Wearable devices offer continuous and often more accurate data; however, they rely on users remembering to charge and wear them, which can lead to decreased usage over time compared to active monitoring. In contrast, active monitoring—based on direct user input—may improve adherence, but the frequency, timing, and content of prompts must be carefully tailored to sustain engagement.

### 3.2 Assessment surveys used

**Figure 3:**
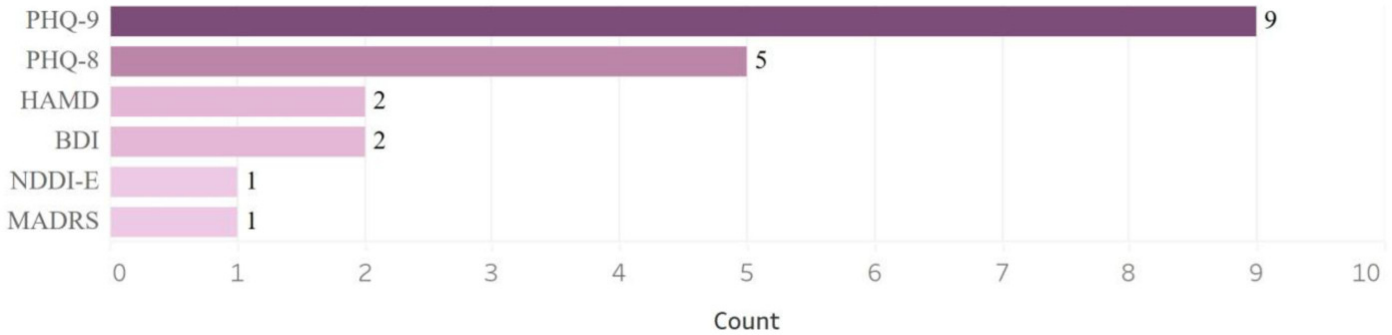
Surveys used to collect data.

The Patient Health Questionnaire-9 (PHQ-9) is one of the most frequently self-administered-questionnaires used to screen for and assess the severity of depression. Our search identified nine studies that used PHQ-9 (14, 15, 19, 21, 28, 30–33) and five studies that utilized PHQ-8 (17, 18, 22, 24, 29). Other questionnaires that were used included the Hamilton Depression Rating Scale (HAMD) (21, 26), the Beck Depression Inventory-II (BDI) (20, 21), the Neurological Disorders Depression Inventory in Epilepsy (NDDI-E) (34), and the Montgomery-Asberg Depression Rating Scale (MADRS) (27, 35) (Figure 3).

### 3.3 Strategies to connect suicidal people to providers

Few studies discussed how suicidal people are connected to the healthcare system. One study describes a national service in Australia, MindSpot, that offers remote mental health screening, assessment, and treatment for adults (32). The questionnaire sent by this program includes questions about suicidal thoughts, with those answering “yes” receiving an immediate on-screen alert that promotes personal safety and provides crisis and emergency service contact details. Additionally, a MindSpot therapist promptly reaches out to conduct a “structured risk assessment” to gather more details on risk factors and, if necessary, refer the individual to emergency services. The questions are sourced from an on-screen form visible only to therapists, ensuring adherence to the risk assessment protocol. As part of the procedure, therapists complete a crisis summary report, which outlines risk and protective factors, documents referrals or other actions taken in response to the assessed risk, and records the outcomes of those actions (32). Another study reported the discussion of a panel on suicide prevention and management (36). The panel was part of a one-day workshop to enhance mental health services for veterans. The panel emphasized the importance of combining call services with onsite follow-ups for building trust and rapport. While call services provide initial contact and support, onsite follow-ups reinforce credibility and strengthen relationships, both on a personal level and within the broader community (36). Another study noted that patients with suicidal intent were referred to appropriate care but did not provide detailed information about the referral process or follow-up outcomes (30).

### 3.4 Barriers of remote mental health screening and proposed solutions

The adoption of RMTs in healthcare has faced several barriers, including technological challenges, user-related factors, legal and ethical concerns, research-related limitations, and difficulties in integrating these technologies into clinical practices. The identified barriers and their subcategories, along with the proposed solutions, are presented in Table 2. More details follow.

**Table 2:** Barriers of implementing remote measurement tools and proposed solutions.

Barriers	Potential solutions
Technological challenges	
Inaccessibility	- Ensuring diverse ways to access mental healthcare - Training in digital tools for elderly
Usability issues	- Simplifying the technology and software - co-design applications with users
Technical malfunction	- Validation and maintenance of software
Intrusiveness	- Transparency i.e., Knowing who gets the data and how it will be used - Sharing only with clinicians
User related challenges	
Stigma	- Inviting all and allowing opting out
Symptom severity	- Easy-to-use design
Patient anxiety related to health monitoring	- Careful design of dashboard that reports progress data to patients
Repeating measures	- Automatic data collection
Attitudes toward technology	- Monitoring patients@ perceptions - Clinicians@ buy-in
Legal and ethical concerns	
Licensing requirements	- Ensuring compliance with hospital policy, state licensure for telehealth, and privacy laws - Ensuring a wide array of providers who are licensed in the state where service is provided.
Liability	- Back-up telephone connections, access to other providers as a safety net.
Fears about privacy and security	- Clear information and opt-out option
Limitation of RMT studies	
Small sample size	- Enhance recruitment and retention
Not separating the impact of RMTs	- Isolating the impact of RMTs by designing studies that focus on specific aspects of RMT
Integrating RMTs into clinical workflows	
Time consuming	- Visualization, training
Varied infrastructures and resources	- A unified system

Technological challenges include inaccessibility, usability issues, technical malfunction, and intrusiveness (1, 6, 23, 37, 38). Studies reported concerns about the price of the technology and access to reliable wi-fi which affect accessibility of the RMTs (6, 38). If accessibility is not addressed, the use of RMT may widen the mental health disparity (1). Ensuring RMTs are not the only entrance level to mental healthcare, and training in digital tools for elderly are essential to minimize inaccessibility (39). Complex technology, regular software updates, “relearning a new operating system,” and technological literacy affect usability (6, 23). Solutions range from simplifying technology and software to codesigning software with users (6, 37). Users reported technical malfunctions such as apps crashing, apps logging out, and difficulties with rescanning QR codes (23). Validation and iterative testing of digital tools ensure they function across different types of devices and settings. Some users were concerned about data sharing and perceived the technology to be intrusive (38). Knowing with whom the data is shared and sharing data only with clinicians might address this issue (38). Wearables were perceived as less intrusive in some studies (23) while others reported invasion of privacy when passive data is reported (15).

User-related barriers include stigma (6, 36, 40), symptom severity (38), “patient anxiety related to health monitoring” (21, 41), not willing to complete repeated measurement surveys (6, 23), and attitudes toward technology (6, 38). To reduce stigma in one study, the invitation to participate in a self-report screening was sent to all personnel in a fire department, and they were allowed to participate or opt out (40). To enhance the adherence of patients with depressive symptoms who may not be able to engage with RMTs, studies suggested easy-to-use-design (38, 39). In addition, training in digital tools for those with low digital literacy has been recommended (39). Careful design of dashboards that report back the collected information to patients is suggested for reducing anxiety generated due to health monitoring (41). Additionally, visualizing patients' health and progress over time facilitates reflection and provides an objective basis for validating improvements in mental health (21). Patients disliked repeating surveys, and they prefer automatic data collections (6). Attitudes toward technology affect the RMT use for all users including providers (38). Some users were concerned about the quality of remote monitoring. As a result, frequent surveys of patients' perception about acceptability of the technology and outcomes are necessary (39). Buy-in by clinicians promotes patients' use of RMTs (6).

Remote assessment of mental health involves legal issues including licensing requirements (i.e., clinicians need to be licensed in the state of the patient), liability (e.g., liability for remote suicide evaluations when technical issues interrupt patient interview) (25), and data security and privacy (6, 39). Proposed solutions for liability related to the interruption include having back-up telephone connections, access to other providers, or other resources (25). Fear about privacy and security are the major barriers of implementing (6, 38, 39). Proposed solutions include ensuring that apps provide clear information on data handling and allowing patients to opt out of data sharing (6, 39).

Small sample size in RMT studies (median sample size = 58) reduce their statistical power and subsequently clinical implementations (38). Lessons learned from large studies, such as the Remote Assessment of Disease and Relapse-Major Depressive Disorder (RADAR-MDD) study, can guide others to enhance recruitments (37) and retention of participants (17). These lessons include co-design with users, ensuring a competent recruitment team with patience and awareness of potential technological barriers, and minimizing participant burden by creating a standardized pathway that guides participants from initial contact to enrolment. In addition, few studies have isolated the impact of RMTs from other factors examined in research projects. Isolating the impact of RMTs is essential for drawing reliable conclusions.

Integrating RMTs into clinical workflows is challenging due to the time-intensive nature of data review and the limited training available for healthcare providers. The sheer volume of data generated by RMTs often exceeds the capacity of clinicians to interpret effectively (6, 41). Automated data visualization tools have emerged as a solution, enabling healthcare providers to quickly interpret trends and anomalies in patient data, reducing mental load and improving efficiency in decision-making (6, 25, 40). Continuous feedback from clinicians has further refined these tools, making sure they align with clinical workflows and complement existing care practices (1, 2, 35). Addressing these challenges involves conducting usability and feasibility studies to identify potential integration issues and adapt tools accordingly. Another barrier for integrating RMT in the clinical setting is the variation in infrastructures and resources across sites (42). A potential solution is a unified clinical informatics system, which has been successfully implemented to streamline screening, assessment, and data integration across pediatric and mental health settings. By incorporating EHR data and patient-reported outcomes, this system has enhanced care coordination for adolescent depression and underscored the need for expanded capabilities, such as patient-facing tools for automated and customized assessments (42).

## 4 Discussion

We conducted a focused literature review about RMTs to identify the most prevalent devices and questionnaires used for measuring mental health factors. Consistent with past studies (1), we found that smartphones were the most popular device for collecting data. Devices were used based on the objective of the studies. Wearable devices provide continuous and more accurate information; however, users need to remember to charge and wear them which may lead to lower use over time relative to active monitoring (1). Active monitoring through direct input from users may increase adherence, but the frequency, timing, and content of prompts should be selected carefully to enhance engagement (1).

The most used questionnaire was the PHQ-9. Many depression questionnaires are available for both the general population and specific subgroups. The choice of questionnaires is guided by the study population. For example, in maternal mental health, the Edinburgh Postnatal Depression Scale (EPDS) and PHQ-9 are frequently used. Additionally, some questionnaires include items on suicidality, which make remote screening challenging—especially if a patient is in acute crisis and requires immediate intervention. On the other hand, not screening for suicidal ideation risks missing critical information and valuable opportunities to intervene. We have successfully used remote screeners that include questions on suicidal ideation and have implemented systems and safety protocols to manage such situations. These include obtaining emergency contact information and the patient's location prior to screening.

We also synthesized barriers to implementing the technologies and the proposed solutions. Our study identified multiple barriers for implementing RMTs including technological challenges, user-related factors, legal and ethical concerns, research-related limitations, and difficulties in integrating these technologies into clinical practices (Table 2). Not all barriers have clear or practical solutions. For example, the repetitive nature of standard mental health questionnaires is a major challenge in maintaining patient engagement. While passive data collection could address this issue (6), it may not be feasible due to patient reluctance, high costs, and the lack of necessity for continuous data collection. Additionally, the data collected from surveys often provide different or additional insight than what is collected from passive devices.

Some hospitals are testing ways to make the user experience more seamless (43–45). For example, partnerships with human factors engineers have been established to study workflow and user interaction with screening tools. This process is iterative and involves qualitative interviews with both staff and patients. The goal is to improve engagement with the tools and reduce the time required to complete questionnaires based on user feedback. In some cases, local hospital teams have been actively involved in developing the software. By leveraging resources already available within the hospital, these efforts aim to ensure the sustainability of software maintenance. This approach also keeps the data within the hospital's systems, making it accessible to patients and protected under HIPAA regulations, rather than becoming the property of external vendors. While this model requires upfront investment from the hospital, it avoids the ongoing maintenance fees associated with commercial products—particularly beneficial in settings with lower patient volumes. Additional efforts include the development of clinician dashboards to monitor patient progress, as well as mobile apps that allow patients to track their own progress. However, adding an app may introduce another step in the process and may not always be perceived as useful by patients. Still, it could provide a secure channel for communication between clinicians and patients. Surveys can also be integrated into the app to assess patient perceptions and gather feedback on the clinical services received.

Most studies identifying barriers relied on interviews with users, including patients and practitioners, and some proposed potential solutions (1, 6, 23, 39). However, no empirical studies have examined the impact of success factors on the effective implementation of RMTs. While the implementation of RMTs in healthcare presents several challenges, the adoption of codesign with users, easy-to-use design, training, the monitoring of patients' perception, clinicians' buy-in, the enhancement of security and privacy measures, multi-method data collection, and automated data analysis can be effective in addressing these barriers, making RMTs a more viable option for remote healthcare, ultimately improving patient engagement and health outcomes. Specifically, security is one of the major challenges in digital mental health, and recent studies are proposing novel frameworks to address it (46).

Although RMTs can enhance access, save costs, collect continuous and detailed data that can be used for self-monitoring and feedback to the healthcare providers (1), and assists in personalized interventions (8, 47–49), it does not replace face-to-face care (41). This review and our experience have shown that patients engage better with RMT if they have been introduced to it by a trusted provider (39). It is also possible that retention and continued engagement would increase if there is a mix of RMT and in person contact including messaging and phone calls. Personal contact prior to use of RMT could influence attitude and increase perceived control thus improving adherence based on the theory of planned behavior (50).

## 5 Limitations

This study has multiple limitations. First, this focused review is designed to answer specific questions we encountered when developing a remote screening system in our hospital which focuses only on screening. For example, our keywords do not include telemedicine, which encompasses a wide range of services such as treatment and care management. Studies that used alternative terms such as e-screening may have been missed due to our limited keywords. Second, unlike systematic literature review, this study does not assess potential biases or the quality of the papers included. The selection of studies was guided by their scope rather than their methodological quality. Future work could benefit from evaluating both scope and quality. Third, we used only three databases to identify relevant papers, and including additional databases could reduce the likelihood of missing relevant papers. Multiple recent systematic literature reviews exist that have addressed these limitations (1, 2, 11, 41).

## 6 Conclusion

Remote Measurement Technologies (RMTs) offer promising avenues for enhancing mental health assessment and care, but their implementation remains complex and context-dependent. Our review identified commonly used devices and questionnaires, as well as multifaceted barriers including technological, user-related, legal, ethical, and integration challenges. While some healthcare settings are actively testing innovative, user-centered solutions—such as incorporating human factors design, building internal systems, and developing mobile apps—widespread adoption still faces hurdles. Our findings underscore the importance of aligning RMT design with patient needs, clinical workflows, and privacy standards. Crucially, successful implementation may depend on hybrid approaches that combine RMTs with trusted human interaction. Future empirical research is needed to evaluate which strategies most effectively support engagement and long-term use, helping realize the full potential of RMTs in mental healthcare.

## References

[1] Walsh AE Naughton G Sharpe T Zajkowska Z Malys M Van Heerden A A collaborative realist review of remote measurement technologies for depression in young people. Nat Hum Behav. (2024) 8(3):480–92. 10.1038/s41562-023-01793-5 38225410 PMC10963268

[2] Goldberg SB Buck B Raphaely S Fortney JC. Measuring psychiatric symptoms remotely: a systematic review of remote measurement-based care. Curr Psychiatry Rep. (2018) 20:1–12. 10.1007/s11920-018-0958-z 30155749

[3] Wies B Landers C Ienca M. Digital mental health for young people: a scoping review of ethical promises and challenges. Front Digit Health. (2021) 3:697072. 10.3389/fdgth.2021.697072 34713173 PMC8521997

[4] Banbury A Roots A Nancarrow S. Rapid review of applications of e-health and remote monitoring for rural residents. Aust J Rural Health. (2014) 22(5):211–22. 10.1111/ajr.12127 25303412

[5] Bardram JE. Remote assessment in healthcare—technologies, methods, benefits, and challenges. PLoS One. (2023) 18(4):e0283945. 10.1371/journal.pone.0283945 37023027 PMC10079064

[6] De Angel V Lewis S White KM Matcham F Hotopf M. Clinical targets and attitudes toward implementing digital health tools for remote measurement in treatment for depression: focus groups with patients and clinicians. JMIR Ment Health. (2022) 9(8):e38934. 10.2196/38934 35969448 PMC9425163

[7] De Angel V Lewis S Munir S Matcham F Dobson R Hotopf M. Using digital health tools for the remote assessment of treatment prognosis in depression (RAPID): a study protocol for a feasibility study. BMJ Open. (2022) 12(5):e059258. 10.1136/bmjopen-2021-059258 35523486 PMC9083394

[8] Kathan A Harrer M Küster L Triantafyllopoulos A He X Milling M Personalised depression forecasting using mobile sensor data and ecological momentary assessment. Front Digit Health. (2022) 4:964582. 10.3389/fdgth.2022.964582 36465087 PMC9715619

[9] Beldjerd MH Lafouge A Giorgi R Le Corroller-Soriano A-G Quarello E. Asynchronous tele-expertise (ASTE) for prenatal diagnosis is feasible and cost saving: results of a French case study. PLoS One. (2022) 17(8):e0269477. 10.1371/journal.pone.0269477 35913933 PMC9342717

[10] You JH Luk SW Chow DY Jiang X Mak AD Mak WW. Cost-effectiveness of internet-supported cognitive behavioral therapy for university students with anxiety symptoms: a Markov-model analysis. PLoS One. (2022) 17(5):e0268061. 10.1371/journal.pone.0268061 35511888 PMC9070891

[11] Bufano P Laurino M Said S Tognetti A Menicucci D. Digital phenotyping for monitoring mental disorders: systematic review. J Med Internet Res. (2023) 25:e46778. 10.2196/46778 38090800 PMC10753422

[12] Adler DA Wang F Mohr DC Choudhury T. Machine learning for passive mental health symptom prediction: generalization across different longitudinal mobile sensing studies. PLoS One. (2022) 17(4):e0266516. 10.1371/journal.pone.0266516 35476787 PMC9045602

[13] Baumel A Edan S Kane JM. Is there a trial bias impacting user engagement with unguided e-mental health interventions? A systematic comparison of published reports and real-world usage of the same programs. Transl Behav Med. (2019) 9:1020–33. 10.1093/tbm/ibz147 31689344

[14] Choudhary S Thomas N Ellenberger J Srinivasan G Cohen R. A machine learning approach for detecting digital behavioral patterns of depression using nonintrusive smartphone data (complementary path to patient health questionnaire-9 assessment): prospective observational study. JMIR Form Res. (2022) 6(5):e37736. 10.2196/37736 35420993 PMC9152726

[15] De Angel V Adeleye F Zhang Y Cummins N Munir S Lewis S The feasibility of implementing remote measurement technologies in psychological treatment for depression: mixed methods study on engagement. JMIR Ment Health. (2023) 10:e42866. 10.2196/42866 36692937 PMC9906314

[16] Klein A Clucas J Krishnakumar A Ghosh SS Van Auken W Thonet B Remote digital psychiatry for mobile mental health assessment and therapy: MindLogger platform development study. J Med Internet Res. (2021) 23(11):e22369. 10.2196/22369 34762054 PMC8663601

[17] Matcham F Carr E White KM Leightley D Lamers F Siddi S Predictors of engagement with remote sensing technologies for symptom measurement in Major depressive disorder. J Affect Disord. (2022) 310:106–15. 10.1016/j.jad.2022.05.005 35525507

[18] Matcham F Leightley D Siddi S Lamers F White KM Annas P Remote assessment of disease and relapse in Major depressive disorder (RADAR-MDD): recruitment, retention, and data availability in a longitudinal remote measurement study. BMC Psychiatry. (2022) 22:136. 10.1186/s12888-022-03753-1 35189842 PMC8860359

[19] Pardes A Lynch W Miclette M Mcgeoch E Daly BP. Use of a mobile health (mHealth) platform for remote assessment of suicidal ideation, depression, and anxiety: a longitudinal retrospective study. Innov Digit Health Diagn Biomark. (2022) 2:8–15. 10.36401/IDDB-21-03

[20] Edward Thomas J Richardson-Vejlgaard R. Remote behavioral sampling for psychological assessment: using interactive technologies to detect depression. Grad Stud J Psychol. (2023) 17:45–55. 10.52214/gsjp.v17i.10919

[21] Thompson A Naidoo D Becker E Trentino KM Rooprai D Lee K. Remote monitoring and virtual appointments for the assessment and management of depression via the Co-HIVE model of care: a qualitative descriptive study of patient experiences. Healthcare. (2024) 12:2084. 10.3390/healthcare12202084 39451498 PMC11508023

[22] White KM Carr E Leightley D Matcham F Conde P Ranjan Y Engagement with a remote symptom-tracking platform among participants with major depressive disorder: randomized controlled trial. JMIR Mhealth Uhealth. (2024) 12:e44214. 10.2196/44214 38241070 PMC10837755

[23] White KM Dawe-Lane E Siddi S Lamers F Simblett S Alacid GR Understanding the subjective experience of long-term remote measurement technology use for symptom tracking in people with depression: multisite longitudinal qualitative analysis. JMIR Hum Factors. (2023) 10:e39479. 10.2196/39479 36701179 PMC9945920

[24] Zhang Y Folarin AA Dineley J Conde P De Angel V Sun S Identifying depression-related topics in smartphone-collected free-response speech recordings using an automatic speech recognition system and a deep learning topic model. J Affect Disord. (2024) 355:40–9. 10.1016/j.jad.2024.03.106 38552911

[25] Godleski L Nieves JE Darkins A Lehmann L. VA Telemental health: suicide assessment. Behav Sci Law. (2008) 26(3):271–86. 10.1002/bsl.811 18548515

[26] Kobak KA Williams JB Engelhardt N. A comparison of face-to-face and remote assessment of inter-rater reliability on the Hamilton depression rating scale via videoconferencing. Psychiatry Res. (2008) 158(1):99–103. 10.1016/j.psychres.2007.06.025 17961715

[27] Kobak KA Williams JB Jeglic E Salvucci D Sharp IR. Face-to-face versus remote administration of the montgomery–asberg depression rating scale using videoconference and telephone. Depress Anxiety. (2008) 25:913–9. 10.1002/da.20392 17941100

[28] Jiang Z, Seyedi S, Griner E, Abbasi A, Bahrami Rad A, Kwon H

[29] Olah J Diederen K Gibbs-Dean T Kempton MJ Dobson R Spencer T Online speech assessment of the psychotic spectrum: exploring the relationship between overlapping acoustic markers of schizotypy, depression and anxiety. Schizophr Res. (2023) 259:11–9. 10.1016/j.schres.2023.03.044 37080802

[30] Contreras C Santa Cruz J Galea JT Chu AL Puma D Ramos L Programmatic implementation of depression screening and remote mental health support sessions for persons recently diagnosed with TB in Lima, Peru during the COVID-19 pandemic. Glob Ment Health (Camb). (2024) 11:e59. 10.1017/gmh.2024.21 38751725 PMC11094547

[31] Mandryk RL Birk MV Vedress S Wiley K Reid E Berger P Remote assessment of depression using digital biomarkers from cognitive tasks. Front Psychol. (2021) 12:767507. 10.3389/fpsyg.2021.767507 34975656 PMC8714741

[32] Nielssen O Dear BF Staples LG Dear R Ryan K Purtell C Procedures for risk management and a review of crisis referrals from the MindSpot Clinic, a national service for the remote assessment and treatment of anxiety and depression. BMC Psychiatry. (2015) 15:1–6. 10.1186/s12888-015-0676-6 26626712 PMC4666146

[33] White KM, Williamson C, Bergou N, Oetzmann C, De Angel V, Matcham F Exploring the definition, measurement, and reporting of engagement in remote measurement studies for physical and mental health symptom tracking: a systematic review (2022)..

[34] Munger Clary HM Snively BM Kumi-Ansu Y Alexander HB Kimball J Duncan P Quality of life during usual epilepsy care for anxiety or depression symptoms: secondary patient-reported outcomes in a randomized trial of remote assessment methods. Epilepsy Res. (2024) 204:107396. 10.1016/j.eplepsyres.2024.107396 38908323 PMC11457121

[35] Giansanti D Siotto M Maccioni G Aprile I. A remote assessment of anxiety on young people: towards their views and their different pet interaction. Healthcare. (2022) 10(7):1242. 10.3390/healthcare10071242 35885769 PMC9320218

[36] Doarn CR Shore J Ferguson S Jordan PJ Saiki S Poropatich RK. Challenges, Solutions, and Best Practices in Telemental Health Service Delivery Across the Pacific Rim—a Summary. New Rochelle, NY USA: Mary Ann Liebert, Inc (2012). 10.1089/tmj.2012.0123 23061646

[37] Oetzmann C White KM Ivan A Julie J Leightley D Lavelle G Lessons learned from recruiting into a longitudinal remote measurement study in major depressive disorder. NPJ Digit Med. (2022) 5:133. 10.1038/s41746-022-00680-z 36057688 PMC9440458

[38] Simblett S Matcham F Siddi S Bulgari V Di San Pietro CB López JH Barriers to and facilitators of engagement with mHealth technology for remote measurement and management of depression: qualitative analysis. JMIR Mhealth Uhealth. (2019) 7(1):e11325. 10.2196/11325 30698535 PMC6372936

[39] Funnell EL Spadaro B Martin-Key NA Benacek J Bahn S. Perception of apps for mental health assessment with recommendations for future design: United Kingdom semistructured interview study. JMIR Form Res. (2024) 8:e48881. 10.2196/48881 38393760 PMC10924263

[40] Wright HM Fuessel-Hermann D Pazdera M Lee S Ridge B Kim JU Preventative care in first responder mental health: focusing on access and utilization via stepped telehealth care. Front Health Serv. (2022) 2:848138. 10.3389/frhs.2022.848138 36925868 PMC10012773

[41] Andrews JA Craven MP Lang AR Guo B Morriss R Hollis C Making remote measurement technology work in multiple sclerosis, epilepsy and depression: survey of healthcare professionals. BMC Med Inform Decis Mak. (2022) 22:125. 10.1186/s12911-022-01856-z 35525933 PMC9077644

[42] Verduin TL. 8.4 Development of an integrated clinical informatics system to support a unified system of care for adolescent depression. J Am Acad Child Adolesc Psychiatry. (2018) 57:S13.

[43] Lagoski M Soghier L Lagata J Shivers M Sadler E Fischer E Mental health support and screening for mood disorders for caregivers in the neonatal intensive care unit: is the call to arms being answered? Am J Perinatol. (2025) 42(03):320–6. 10.1055/a-2353-0993 38925161

[44] Sadjadpour F Hosseinichimeh N Jarvis L Perazzo S Soghier L. A participatory approach to enhance screening system for perinatal mood and anxiety disorder in critical care settings. Syst Res Behav Sci. (2025). 10.1002/sres.3173

[45] Sadler EM Okito O Soghier L. Addressing caregiver mental health in the neonatal ICU. Curr Opin Pediatr. (2023) 35(3):390–7. 10.1097/MOP.0000000000001242 36974450

[46] Georgiou D Katsaounis S Tsanakas P Maglogiannis I Gallos P. Towards a secure cloud repository architecture for the continuous monitoring of patients with mental disorders. Front Digit Health. (2025) 7:1567702. 10.3389/fdgth.2025.1567702 40661652 PMC12256522

[47] Abbas A Sauder C Yadav V Koesmahargyo V Aghjayan A Marecki S Remote digital measurement of facial and vocal markers of Major depressive disorder severity and treatment response: a pilot study. Front Digit Health. (2021) 3:610006. 10.3389/fdgth.2021.610006 34713091 PMC8521884

[48] Hornstein S Zantvoort K Lueken U Funk B Hilbert K. Personalization strategies in digital mental health interventions: a systematic review and conceptual framework for depressive symptoms. Front Digit Health. (2023) 5:1170002. 10.3389/fdgth.2023.1170002 37283721 PMC10239832

[49] Wanniarachchi VU Greenhalgh C Choi A Warren JR. Personalization variables in digital mental health interventions for depression and anxiety in adolescents and youth: a scoping review. Front Digit Health. (2025) 7:1500220. 10.3389/fdgth.2025.1500220 40444184 PMC12119569

[50] Godin G Kok G. The theory of planned behavior: a review of its applications to health-related behaviors. Am J Health Promot. (1996) 11(2):87–98. 10.4278/0890-1171-11.2.87 10163601

